# Nursing diagnosis identification by nurses in burn wards: A descriptive cross‐sectional study

**DOI:** 10.1002/nop2.470

**Published:** 2020-03-10

**Authors:** Mohammad Khajehgoodari, Mojgan Lotfi, Vahid Zamanzadeh, Leila Valizadeh, Parisa Khalilzad

**Affiliations:** ^1^ Department of Medical Surgical Nursing Faculty of Nursing and Midwifery Sina Hospital Tabriz University of Medical Sciences Tabriz Iran; ^2^ Department of Medical Surgical Nursing Faculty of Nursing and Midwifery Tabriz University of Medical Sciences Tabriz Iran; ^3^ Department of Pediatric Nursing Faculty of Nursing and Midwifery Tabriz University of Medical Sciences Tabriz Iran; ^4^ Behboud Hospital Tabriz University of Medical Sciences Tabriz Iran

**Keywords:** nursing diagnosis burn unit, nursing record

## Abstract

**Aim:**

To identify and document patients' care needs, it is vital to provide quality care services. This study was aimed to describe care needs derived from records of patients with burn and to evaluate whether nurses employed the North American Nursing Diagnosis Association classification to formulate patients' care needs.

**Design:**

A descriptive cross‐sectional study.

**Methods:**

In this study using the convenient sampling method, 430 nursing records reviewed in the burn wards. Data were collected using Gordon's checklist. The validity of the checklist assessed by content validity and the reliability of them calculated with inter‐rater and internal consistency. Data analysed by SPSSv.24.

**Results:**

The mean number of diagnoses per record was 1.94. The most frequent diagnosis was in the domain of Safety/Protection and the top two prevalent nursing diagnoses in Sina hospital were a risk for infection and risk for falls. From all of the detected diagnostic, about 83% were determinedly not related to one of 247 labels of the North American Nursing Diagnosis Association. Given that nurses provide nursing care as requested by physicians and patient care needs are not assessed and recorded by them, it can be concluded that there was no nursing thinking behind their nursing care.

## 1 INTRODUCTION

Nurses must properly identify and document patients' care needs to provide quality care services and to evaluate the nursing care delivered (Müller‐Staub, 2009). This care needs or patient health problems are identified by nurses and documented as nursing diagnoses in the records as well as in the nursing Kardex (Crabtree, Howard, & El‐Mallakh, 2009; Potter & Perry, 2017). The application of nursing diagnoses in clinical settings is important to promote evidence‐based nursing care (Carpenito‐Moyet, 2010; Müller‐Staub, 2009). Nurses must use comprehensive assessment forms to achieve correct nursing diagnoses, and they have professional communication with patients. To accomplish the assessment, nurses must understand bio‐psychosocial concepts, people's physical and spiritual needs, the growth/evaluation, the pathophysiology of diseases, family systems and culture and the value of patient beliefs (Potter & Perry, 2017).

To ensure that patients' care needs are met, nurses need to make systematic observations of patients, communicate verbally and non‐verbally, listen well, create effective relationships, build trust and conduct assessments through interviews or physical examinations (Potter & Perry, 2017).

Nursing assessment and diagnosis are the first two steps of the nursing process in nursing science that allow nurses to identify patient problems (Potter & Perry, 2017). Accurately assessing patients' care needs and precise formulation of nursing diagnoses is essential because of the nursing diagnoses as a basis for the beginning point for planning patient outcomes and effective nursing interventions (Gordon, 2008).

To perform a suitable nursing diagnosis, a nurse needs knowledge and skills such as clinical reasoning, critical thinking, data analysis and ability to integrate all information for conclusions an understanding of potential conditions, characteristics of the possible conditions, standard measurements used to detect problems and mechanisms of disease processes (Herdman & Kamitsuru, 2019; Potter & Perry, 2017).

A precise nursing diagnosis describes a patient's problem, related factors (aetiologies) and defining characteristics (signs and symptoms) in an unequivocal, unambiguous language (Gordon, 2008; Lunney, 2008). These diagnoses are published by the North American Nursing Diagnosis Association‐International (NANDA‐I) every 2 years. NANDA‐I latest Nursing Diagnosis list had 247 nursing diagnoses published in the year 2018 (Herdman & Kamitsuru, 2019).

Numerous studies have been conducted in different countries with the aim of identifying patient care needs in nursing records based on nursing diagnoses such as patients in Dutch hospitals (Paans & Muller‐Staub, 2015), nursing diagnosis in a hospital in Iceland (Thoroddsen & Thorsteinsson, 2002), nursing diagnoses in elderly residents of a nursing home (Guler, Eser, Khorshid, & Yucel, 2012), geriatric rehabilitation setting (Heering, 2010), critical care patients (Gordon & Hiltunen, 1995), patients with cardiac insufficiency (Matos, Guimarães, Brandão, & Santoro, 2012) and oncology patients in the dying phase (van der Werff, Paans, & Nieweg, 2012). But according to our knowledge, no study has addressed the prevalence and accuracy of nursing diagnoses in relationship to the NANDA‐I theoretical framework in general Iran hospitals. Most studies on nursing documentation in Iran have focused more on the quality of nursing documentation such as recording vital signs assessment, medication treatment, intake and output of fluids (Aghdam, Jasemi, & Rahmani, 2009; Ghazanfari, Sheykhpour‐khani, & Haghdoost, 2009; Lotfi et al., 2019b; Tabrizi, Rahmani, Jafarabadi, Jasemi, & Allahbakhshian, 2016). A review of the literature shows that potential shortcomings in the quality of nursing records in Iran. Several studies have been performed in the internal‐medicine wards, surgical wards and intensive care units and these studies have shown substantial instances of lack documentation or incomplete documentation of executed tasks in different hospital wards (Aghdam et al., 2009; Ghazanfari et al., 2009; Kahouei et al., 2014; Tabrizi et al., 2016). So far no study has been conducted on the care needs of burn patients, in other words, the least attention has been paid to the care needs of these patients.

Burn injuries cause disabilities, psychological problems, complications like scarring, infection, breathing problems, low blood volume, dangerously low body temperature, bone and joint problems that may result in multi‐organ failure, sepsis and which eventually leads to death (Mirmohammadi et al., 2012; Stoddard, Ryan, & Schneider, 2014). Though nowadays with the development of specialized burn centres and associated advances in treatment, has been decreased mortality rates these patients, burns still lead to complex metabolic changes that can adversely affect the whole body system (Brusselaers, Monstrey, Vogelaers, Hoste, & Blot, 2010; Herndon, 2018).

In Iran, 150,000 burns occurred each year, with an annual death of 3,000 individuals (Saberi, Fatemi, Soroush, Masoumi, & Niazi, 2016). Burns comprise 5% of the total number of accidents in Iran and account for 6% of all deaths from accidents in this country (Roham, Anbari, Fatemi, & Momeni, 2017).

Although the NANDA‐I taxonomy taught in basic nursing education in Iran, but there is no study of how well nursing records are written according to this taxonomy. This situation may lead nurses to ignore the problems and care needs of the patient. Hence, this study was aimed to describe care needs derived from records of patients with burn and to evaluate whether nurses employed the NANDA‐I classification to formulate patients' care needs.

## 2 METHODS

### 2.1 Design

It was a descriptive cross‐sectional study carried out in the form of a research proposal approved by Tabriz University of Medical Sciences from September 2018–December 2018. Patients care needs were reviewed by conducting data analysis of nursing records and using the Gordon health performance checklist.

### 2.2 Setting and participants

After receiving permission from university officials and management and obtaining the ethical code with number IR.TBZMED.REC.1397.170 from Tabriz University of Medical Sciences, nursing records in Sina hospital were obtained to assessing in the study. Sina hospital as an educational centre affiliated to Tabriz University of Medical Sciences is the Northwest Burn Center of Iran and has 78 active beds for patients with burn (the women's burns ward has 19 beds, the men's and paediatric burns ward each has 18 beds, reconstruction wards has 14 beds and BICU has 9 beds). The families speak Persian, Azerbaijani and Turkish.

To calculate the sample size, using G*Power with 0.80 power at the 0.05 alpha level, the average hospitalization of 1,100 patients with burns annually in this hospital and an estimated effect size of 0.30 were considered and the sample size of 425 was required. Since all patients hospitalized during the 4 months were 430 people; therefore, all nursing records of the patients with burn (430 records) were used in this study.

Using the convenient sampling method, all nursing records were obtained in this study at the time of discharge patients with burns. The inclusion of nursing records was based on two criteria: (a) the patient's length of stay (at least 3 days) and (b) the patient's written informed consent to study their nursing documentation (parental consent of children with burns was also required).

### 2.3 Data collection and procedures

Data were collected using the Gordon functional health Patterns checklist (GFHPC). The checklist had been developed in accordance with the literature by the researchers in the study. All NANDA‐I nursing diagnoses list 2018–2020 are divided into 13 domains of Gordon's functional health patterns (Herdman & Kamitsuru, 2019). The purpose of NANDA‐I Taxonomy as a clinical judgement concerning a human response to health conditions & life processes, or vulnerability for that response is to provide a recognized and clinically beneficial classification for the achievement of a standardized description of nursing diagnoses (Herdman & Kamitsuru, 2019). As of 2018–2020, NANDA‐I taxonomy had 5 levels: 7 domains, 34 classes, 540 outcomes with multiple Indicators and Measurement of Outcomes (Moorhead, Johnson, Maas, & Swanson, 2018). In general, it has approved 247 nursing diagnoses (Herdman & Kamitsuru, 2019). This classification system is widely used in several countries (Di Mauro, Vanalli, Alberio, & Ausili, 2018; Guler et al., 2012; Paans & Muller‐Staub, 2015; Semachew, 2018). In our country, it has been a current trend to use in a few hospitals for a short time and commonly in education settings. In our study, we designed a checklist based on 13 domains which include comprehensive information such as health promotion, elimination and exchange, nutrition, activity and rest, perception and cognition, self‐perception, role relationship, sexuality, coping and stress tolerance, life principles, safety and protection, comfort and growth and development (Herdman & Kamitsuru, 2019). The instrument was developed to measure the accuracy of the assessment at admission and during hospitalization and nursing diagnoses (problem label, aetiologies or related factors and signs and symptoms). The GFHPC was integrated to strengthen and as a guide to define nursing diagnosis according to Nursing Diagnoses Grouped in NANDA‐I list 2018–2020. This checklist included 26 items with yes/no questions. The minimum score is 26, and the maximum score is 52. A higher score reflects better nursing documentation (Table 1).

**Table 1:** The checklist based on Gordon's functional health patterns (GFHPC)

Item		Yes	No
1	Has the nurse assessed the domain of Patient Health Promotion?		
2	Are nursing diagnoses recorded for this domain? If yes, Please write it in front of the question		
3	Has the nurse assessed the domain of Patient Nutrition?		
4	Are nursing diagnoses recorded for this domain? If yes, Please write it in front of the question		
5	Has the nurse assessed the domain of Patient Elimination and exchange?		
6	Are nursing diagnoses recorded for this domain? If yes, Please write it in front of the question		
7	Has the nurse assessed the domain of Patient Activity/rest?		
8	Are nursing diagnoses recorded for this domain? If yes, Please write it in front of the question		
9	Has the nurse assessed the domain of Patient Perception/cognition?		
10	Are nursing diagnoses recorded for this domain? If yes, Please write it in front of the question		
11	Has the nurse assessed the domain of Patient Self‐perception?		
12	Are nursing diagnoses recorded for this domain? If yes, Please write it in front of the question		
13	Has the nurse assessed the domain of Patient Role relationship?		
14	Are nursing diagnoses recorded for this domain? If yes, Please write it in front of the question		
15	Has the nurse assessed the domain of Patient Sexuality?		
16	Are nursing diagnoses recorded for this domain? If yes, Please write it in front of the question		
17	Has the nurse assessed the domain of Patient Coping/stress tolerance?		
18	Are nursing diagnoses recorded for this domain? If yes, Please write it in front of the question		
19	Has the nurse assessed the domain of Patient Life principles?		
20	Are nursing diagnoses recorded for this domain? If yes, Please write it in front of the question		
21	Has the nurse assessed the domain of Patient Safety/protection?		
22	Are nursing diagnoses recorded for this domain? If yes, Please write it in front of the question		
23	Has the nurse assessed the domain of Patient Comfort?		
24	Are nursing diagnoses recorded for this domain? If yes, Please write it in front of the question		
25	Has the nurse assessed the domain of Patient Growth/development?		
26	Are nursing diagnoses recorded for this domain? If yes, Please write it in front of the question		

The content and face validity of the GFHPC were confirmed by a panel of experts consisting of 7 faculty members of Tabriz University of Medical Sciences and 5 experienced nurses in burn care. Some minor changes were applied according to experts' recommendations. The internal consistency of the GFHPC (Kuder–Richardson reliability coefficient) was 0.83 and the inter‐rater reliability (kappa coefficient) varied between 0.80–0.87.

Each patient record was assessed by 2 researchers according to the GFHPC independently. 5 head nurses served as reviewers, and these reviewers were taught how to evaluate nursing documentation according to the NANDA‐I classification for 2 weeks.

### 2.4 Statistical analysis

Data were analysed by descriptive statistics using SPSS 24 software. Frequencies and percentages scores of diagnostic findings were calculated, as well as means and standard deviations.

## 3 RESULTS

Over the 4‐month study period, 430 patients were hospitalized in five burn wards and nursing records of these patients were assessed at the time of discharge. The greatest mean number of nursing diagnoses per documentation was discovered in the BICU, *N* = 3.08 and the lowest in the paediatric burns ward, *N *= 0.63. Table 1 shows the prevalence of patients' admission and nursing diagnosis in 5 wards in the hospital.

Based on the review of 430 nursing records, 836 nursing diagnoses were found. The mean number (*SD*) of diagnoses per record was 1.94 (0.8). The most frequent diagnosis was in the domain of Safety/Protection, and the top two prevalent nursing diagnoses in Sina hospital were a risk for infection (*N* = 193) and risk for falls (*N* = 174). 80 nursing records contained 4 diagnoses, which was the maximum number of determined diagnoses and 65 nursing records contained no diagnosis (Table 2). The review of nursing records revealed the risk for falls determinate for patients without any special sign or symptoms by nurses.

**Table 2:** Number of patients admitted and nursing diagnosis (ND) detected in five burn wards during the 4‐month study

Ward	*N*	%	Number of ND
Women's burns ward	85	19.76	172
Men's burns ward	142	33.02	246
Paediatric burns ward	73	17	115
Reconstruction wards	76	17.67	137
BICU (Burns Intensive Care Unit)	54	12.55	166
Total	430	1	836

Note

*N*, Number of patients; %, Frequency percentage.

All nursing diagnoses are identified at the time of the initial nursing assessment (assessment of admission), but during the hospitalization stay, no nursing diagnosis was identified for the patients. From all of the detected diagnostic labels, about 83% were determined not related to one of 247 NANDA‐I problem labels. There was no supervision to record nursing diagnoses or no follow‐up to continue nursing care based on nursing diagnoses (Table 3). There was no relationship between descriptive characteristics, signs and symptoms with all nursing diagnoses. In other words, these documented nursing diagnoses were without regard to the actual problems of the patients (Table 4).

**Table 3:** The distribution of nursing diagnoses most commonly identified in the patients with burn

Domain and ND categories	*N*	%
Domain 1		
Health promotion	0	–
Domain 2		
Nutrition		
**Imbalanced nutrition: less than body requirements**	69	8.25
Domain 3		
Elimination and Exchange	0	–
Domain 4		
Activity/Rest	0	–
Domain 5		
Perception/Cognition		
**Deficient knowledge**	112	13.39
Domain 6		
Self‐perception	0	–
Domain 7		
Role relationships	0	–
Domain 8		
Sexuality	0	–
Domain 9		
Coping/Stress Tolerance		
**Anxiety**	134	16.03
Domain 10		
Life principles	0	–
Domain 11		
Safety/Protection		
**Risk for infection**	193	23.09
**Risk for falls**	174	20.81
Domain 12		
Comfort		
**Acute pain**	154	18.43
Domain 13		
Grow/evaluation	0	–

Note

*N*, Number of patients; %, Frequency percentage.

**Table 4:** Accuracy and precision of nursing diagnoses in burn wards

Item	*N*	%
Identifying nursing diagnoses after initial nursing assessment	836	100
Identifying nursing diagnoses during hospitalization	0	0
Diagnostic issues not related to NANDA‐I classification	693	82.90
Nursing diagnosis according to NANDA‐I classification	143	17.10
Identification of causes of problems	20	2.39
Problems arranged by priority	7	0.83
Follow‐up on nursing diagnoses in subsequent shifts	0	0

Note

*N*, Number of patients; %, Frequency percentage.

Based on valid and comprehensive references (Herndon, 2012; Paul, Day, & Williams, 2016; Wolf, Cancio, & Pruitt, 2018), the burns care categorized into two main phases (emergency and acute phases). The emergency phase also referred to as resuscitative phase, which begins with the onset of burn injury and may be completely bypassed in the first 24–48 hr postburn injury. The acute phase starts as soon as the emergency phase completely bypassed and it will continue until wound closure. The duration of this phase may take 2 weeks or more. The study found that all identified nursing diagnoses were recorded in the emergency phase because these nursing diagnoses were recorded in the early hours of admission in the inpatient ward. There was not any statistically significant difference between clinical characteristics with nursing diagnosis (Table 5).

**Table 5:** General clinical characteristics of study participants and nursing diagnosis (ND) detected in five burn wards during the 4‐month study

Clinical characteristics	*N* (%)	Number of ND (%)	*P* value
Depth of burn			
Grade II	147 (34.18)	279 (31.70)	.62
Grade III	125 (29.06)	292 (34.93)	
Grade II and III	158 (36.74)	265 (33.37)	
Inhalational injuries	95(22.09)	73 (8.73)	.12
Burns severity			
<10	115 (26.75)	186 (22.24)	.06
10–20	123 (28.60)	223 (26.67)	
21–30	107 (24.88)	212 (25.36)	
>30	85 (19.77)	215 (25.72)	
Location of Burn			
Upper limb	94 (21.86)	135 (16.15)	.23
Lower limb	101 (23.49)	148 (17.71)	
Head and neck	63 (14.65)	139 (16.63)	
Body	71 (16.52)	138 (16.50)	
Upper & lower limb	56 (13.02)	133 (15.91)	
Upper & lower limb and body	45 (10.46)	143 (17.10)	

Note

*N*, Number of patients; %, Frequency percentage.

## 4 DISCUSSION

Nursing records are important in professional nursing practice (Okaisu, Kalikwani, Wanyana, & Coetzee, 2014). This documentation is a piece of the nursing activity of vital importance (Papathanasiou, Kotrotsiou, & Bletsa, 2014). To improve the quality of patient care, nurses should use nursing diagnoses with a systematic assessment according to special patterns and should help the patients with burn to health promotion, accelerate recovery and use of the maximum current potential. Based on NANDA‐I classification, patients' care needs were formulated according to NANDA‐I labels (Moorhead et al., 2018). In this study, we found that nurses in the burn clinical setting were not familiar with this classification. The NANDA‐I 2018–2020 classification contained 247 validated nursing diagnoses (Herdman & Kamitsuru, 2019). Of these NANDA‐I diagnoses, only 6 labels were evident in nursing records. It may be concluded that the other 241 nursing diagnoses were not employed in burn wards.

In the studies of Guler et al. (2012) revealed that 165 nursing diagnoses distributed across the several domain classes of the taxonomy used were identified. In the study of Paans and Muller‐Staub (2015), 47 labels were evident in nurses' patient documentations. In other studies, the prevalence of nursing diagnoses in nursing records was also used more than 20 nursing diagnoses were reported (Thoroddsen & Thorsteinsson, 2002; Vasconcelos, Araújo, Moreira, & Lopes, 2007). Certainly, cultural differences are influential in assessing and documenting the prevalence of nursing diagnoses. In Iran, for three reasons, the nursing diagnoses are not conducted extensively and continuously at the hospitals or performed very poorly: low proficiency of faculty members and nurses from the concept of the nursing diagnoses, lack of necessary infrastructure and lack of supporting nursing institutions and managers (Lotfi et al., 2019b).

In Iran, so far no study has been conducted to review nursing diagnoses documented in nursing records. Studies on nursing documentation in Iran have focused more on the quality of nursing documentation. These studies have reported the quality of nursing documentation was unsuitable and did not contain necessary information (Tabrizi et al., 2016; Vafaei, Manzari, Heydari, Froutan, & Farahani, 2018). These studies reviewed the nursing records of patients based on nursing activities though they did not pay attention to patient care needs and the documented nursing diagnoses.

The results of this study showed that the assessment and nursing diagnoses documented in the nursing records were made only at the time of admission. The documented nursing diagnoses also did not match the patients' care needs, and most of the patients' care needs were not considered. The most important reason for this issue was the incomplete initial nursing assessment form. This form did not have the appropriate scientific content to guide nurses for identifying patients' needs. In a descriptive study conducted by Sumitra, the results showed that 50% of nursing assessment forms did not contain complete information which was similar to our results (Sumitra, 2001). Other studies on nursing assessment documents and consistent with this study showed that the documentation process was not capable of capturing the information from nursing diagnoses (Hidayat & Kes, 2015; Noorkasiani, Gustina, & Maryam, 2015). This study demonstrates that no effort is being made to develop a comprehensive assessment form in accordance with the NANDA. Therefore, documentation and implementation of nursing diagnoses have not occurred according to the NANDA in Iran. Due to the weakness of the nursing assessment form, the quality of communication between nurses and patients may not be appropriate (Lotfi, Zamanzadeh, Valizadeh, & Khajehgoodari, 2019). As a result, patients' care needs will not be identified.

There was no documentation of assessment done by nurses. Most of the nursing documentation recorded in the records were related to the procedures that physicians requested from nurses. There is no evidence that the nurse has examined the patient and it has extracted the care needs of the patients. These findings indicate that nurses pay less attention to patient care needs which is not consistent with Guler et al. (2012) and Paans and Muller‐Staub (2015) studies. Dependency on medical diagnoses and their implementation by nurses does not provide enough information to accurately diagnose a patient from a nursing perspective, also, keeps nursing away from the professional. The concurrent use of medical diagnosis and nursing diagnoses allows for a better description of a patient's actual situation to be made and provides a better basis for decisions about adequate interventions (Hidayat & Kes, 2015). The nursing assessment form in burn wards is ineffective practically, most in these wards use the doctor's assessment rather than the nurse's assessment.

The results of this study demonstrate that, of the total diagnostic labels reviewed, only about 17% of the labels were determinedly related to one of 247 NANDA‐I classification, while in Guler et al study in Turkey, Paans & Muller‐Staub in the Netherlands and Thoroddsen, A., & Thorsteinsson, H in Iceland, they were about 54%, 94.1% and 60%, respectively (Guler et al., 2012; Paans & Muller‐Staub, 2015; Thoroddsen & Thorsteinsson, 2002). Understanding the NANDA classification is lack among nurses in burn wards. Therefore, the level of awareness of nurses should be increased by proper and comprehensive assessment methods as well as the identification of nursing diagnoses related to NANDA‐I classification in these wards. Knowledge obtained through sources such as the Gordon functional health patterns assessment assists nurses in achieving proper nursing diagnosis (Spenceley, O'Leary, Chizawsky, Ross, & Estabrooks, 2008). The purpose of review nursing records based on the Gordon functional health patterns assessment and NANDA was to attain greater accuracy in nursing diagnoses.

### 4.1 Study limitations

The data from this study provide an initial description of patients' care needs and nursing diagnoses in the Northwest Burn Center of Iran. The results of the research may not be generalized beyond nursing documentation from other health centres. The influence of instruments was not assessed in current study. This study focuses on the emergency postinjury period until the first days of the acute period (time of discharge) and cannot extend to other stages of burn care. It is recommended that the study be replicated using a larger sample size in a variety of health centres.

## 5 CONCLUSION

A review of the nursing records revealed that nurses do not document patients' care needs. They provide nursing care as requested by physicians. In other words, the nursing records are based on the procedures that physicians have asked. Given that nurses provide nursing care as requested by physicians and patient care needs are not assessed and recorded by nurses, it can be concluded that there is no nursing thinking behind their nursing care. The lack of a proper documentation system for nurses is evident in this study. Moreover, nurses' awareness of nursing diagnoses is very poor. In fact, there was no training provided for them in this regard.

## CONFLICT OF INTEREST

None.

## AUTHOR CONTRIBUTIONS

MKH and ML: Contributed to conception and study design, data collection, analysis, interpretation, manuscript writing, reviewing, and revising it. VZ and LV: Contributed to conception and study design, data interpretation, reviewing, and revising the manuscript. PKH and MKH: Contributed to data interpretation, manuscript writing, reviewing, and revising it. All authors read and approved the final manuscript.

## ETHICAL APPROVAL

Approval code of ethics with number: IR.TBZMED.REC.1397.170.
